# Cross-Sectional Associations of Dietary Pattern, Physical Activity with Hypertension Among Adults in Southwestern Nigeria

**DOI:** 10.3390/nu18172825

**Published:** 2026-08-28

**Authors:** Animashaun Adeola Faidat, Aliya Yijiati, Xingyu Yu, Yuanyuan Jiang, Asaolu Segun, Zhini He

**Affiliations:** 1 Food Safety and Health Research Center, School of Public Health, Southern Medical University, Guangzhou 510515, China; animashaunadeola3@gmail.com (A.A.F.); all99103@163.com (A.Y.); moyuwan982@163.com (X.Y.); jiangyuanyuan0319@126.com (Y.J.); 2 Guangdong Provincial Key Laboratory of Tropical Disease Research, Guangzhou 510515, China; 3 Department of Biochemistry, Ekiti State University, Ado-Ekiti 362001, Nigeria; asaolu29@gmail.com

**Keywords:** dietary patterns, hypertension, physical activity, principal component analysis, cross-sectional study, Nigerian adults

## Abstract

Background: Hypertension and unhealthy traditional diets often coexist among Nigerian adults, but limited regional evidence clarifies how local dietary patterns correlate with hypertension prevalence, leading to fragmented, culturally insensitive prevention guidelines. Objectives: This cross-sectional analysis examined cross-sectional associations between PCA-derived dietary patterns and physical activity levels and hypertension prevalence among adults residing in Oyo and Kwara States, southwestern Nigeria. Methods: A total of 608 eligible adults completed a culturally validated 7-day Food Frequency Questionnaire (FFQ) and Global Physical Activity Questionnaire (GPAQ). Standardized single-visit blood pressure measurements defined hypertension per WHO criteria. Robust cluster-adjusted Poisson regression was used to estimate prevalence ratios (PR) and 95% confidence intervals (95% CI, all two-sided *p* < 0.05). Results: The overall prevalence of hypertension was 24.34%, with significantly higher prevalence among males (29.78%) compared to females (16.67%). Four distinct dietary patterns were extracted via Varimax-rotated PCA: diversified traditional pattern, typical traditional pattern, fruits and vegetables pattern, and fats and soft drinks pattern. After full covariate adjustment, highest adherence to the typical traditional pattern was significantly associated with higher hypertension prevalence (PR = 1.642, 95% CI: 1.069–2.252, *p* = 0.024). In contrast, maximum adherence to the fruit and vegetable pattern was associated with lower hypertension prevalence (PR = 0.408, 95% CI: 0.258–0.646, *p* < 0.001). No significant cross-sectional associations were detected for diversified traditional or fats and soft drinks patterns. The crude positive activity–hypertension association turned non-significant after adjusting for all dietary patterns. Conclusions: Distinct local dietary patterns displayed consistent cross-sectional associations with hypertension prevalence in this population, while physical activity showed no independent correlation with hypertension after accounting for habitual diet.

## 1. Introduction

Hypertension represents one of the most widespread preventable health burdens worldwide, affecting over 1.4 billion adults globally and contributing to approximately 16% of all premature deaths, while non-communicable diseases collectively account for 74% of global mortality [1,2]. National pooled estimates place hypertension prevalence among Nigerian adults aged 30–79 years between 28.9% and 36.1% [3,4]. Yet low community awareness and uneven access to routine clinical screening leave most cases undetected and poorly managed [5,6].

Earlier research exploring diet and hypertension frequently isolated single nutrients or individual food items, which oversimplifies the complexity of real-world eating habits and overlooks synergistic effects among dietary components [7,8,9]. Dietary pattern analysis, in contrast, provides a holistic approach by evaluating the overall combination of foods consumed, offering more relevant insights for developing effective prevention and management strategies [10,11]. International applications of this method have identified protective patterns such as the DASH diet, while Westernized high-fat, high-sugar patterns consistently correlate with increased hypertension risk [12,13,14].

Within West Africa, including Nigeria, existing observational data reveal that high intakes of salt, red meat, processed foods, and alcohol are associated with elevated hypertension odds, whereas fruit and vegetable consumption appears protective [15,16]. We now clarify that existing Nigerian PCA dietary pattern studies cover broad national cohorts [15,17,18], but no published work has focused exclusively on community residents of Oyo and Kwara States, nor jointly analyzed the combined interplay between four local dietary patterns and stratified physical activity levels in relation to hypertension. This regional and multi-exposure joint analysis is clearly marked as the core novelty of this work.

This cross-sectional study therefore aims to address these critical regional research gaps. We first estimated hypertension prevalence among adults from Oyo and Kwara States, then extracted dominant dietary patterns via PCA, quantified weekly physical activity volumes, and assessed independent and combined associations between these lifestyle exposures and hypertension status. The findings will generate locally relevant evidence to inform culturally appropriate dietary guidelines, public health policies, and community-based interventions, ultimately contributing to the reduction in hypertension-related morbidity and mortality in Nigeria.

## 2. Materials and Methods

### 2.1. Study Design and Participant Recruitment

This cross-sectional study was conducted from November to December 2025 across four selected Local Government Areas (LGAs) from Oyo and Kwara States, southwestern Nigeria. The full sampling frame included all 33 LGAs of Oyo State and 16 LGAs of Kwara State. A multistage stratified cluster random sampling design was implemented with four sequential recruitment stages: (1) Two LGAs were randomly selected per state via random number generation, generating four target LGAs total. (2) Each selected LGA was split into urban and rural strata; 2–3 towns were selected per stratum using probability proportional to size (PPS), resulting in five towns per LGA (20 towns overall). (3) All community/street registers were obtained from local administrative offices; five streets/communities were randomly sampled in each town via simple random sampling. (4) Systematic household sampling was adopted on each selected street, with a random starting point; investigators enrolled 10 eligible adults per town. Within multi-adult households, one participant was randomly selected.

Eligibility and Exclusion Criteria are as follows. (1) Inclusion: Aged ≥ 18 years, permanent local resident, willing to provide signed written informed consent. (2) Exclusion: Active acute severe illness, diagnosed non-hypertension chronic organ disease, current pregnancy, self-reported intentional short-term dietary modification. Participants with diagnosed hypertension or regular antihypertensive medication use were fully retained in the sample (to avoid prevalence underestimation and selection bias).

The sample size was calculated using the correlation analysis formula:

n = [(Z_α_ + Z_β_)/C]^2^ + 3

Two-sided α = 0.05 (Z_α_ = 1.96); statistical power = 80% (β = 0.20, Z_β_ =0.8416). The expected correlation coefficient r = 0.12 was extracted from a similar study [19]. Fisher’s z-transformation: C = 0.5 × ln[(1 + r)/(1 − r)] = 0.1206. Unadjusted minimum *n* = 543. Adding a 10% contingency for non-response or incomplete data (*n* = 54) gave a final target sample size of 597. The final valid analytical sample reached 608 participants.

Ethical approval was issued by the Oyo State Ministry of Health (Approval Reference Number: AD 13/479/642c, Approval Date: 4 November 2025). All participants provided signed written informed consent, as formally approved by the ethics committee.

### 2.2. Data Collection Procedures

Two distinct data were collected in this study: interview-based questionnaire responses and standardized clinical physical measurements. A structured questionnaire captured sociodemographic characteristics (age, sex, education, income, occupation), lifestyle factors, medical history, and family history of hypertension. Dietary intake was assessed using a culturally adapted 7-day Food Frequency Questionnaire (FFQ) comprising 100 food items. All 100 discrete food items were manually aggregated into 17 preliminary broad food categories, based on similarities in macronutrient profiles, local cooking modes and regional food composition references (e.g., swallow-based foods, legumes, cereals, red meat, white meat, fruits, vegetables, and beverages). The full matching list of individual food items within each of the 17 categories is provided in Supplementary Table S1.

Physical activity was measured using the Global Physical Activity Questionnaire (GPAQ). Four activity domains were quantified: occupational work activity, commuting travel activity, recreational movement, and sedentary behavior. Weekly total MET-minutes were calculated by multiplying activity MET coefficients × duration × weekly frequency. Participants were classified strictly by WHO official cut-offs: (1) low activity: <600 MET-min/week; (2) moderate activity: ≥600 MET-min/week; and 3) high activity: ≥3000 MET-min/week OR ≥3 days weekly of vigorous activity reaching ≥1500 MET-min/week. Continuous weekly MET-min values were used for partial correlation analysis; categorical tiers were applied in regression models.

Blood pressure was measured using a validated digital sphygmomanometer (Omron HEM-7430) [20]. The device was calibrated at launch and monthly throughout fieldwork. Measurement standards: seated position with feet flat, 5 min quiet pre-rest, right upper arm at heart level, individually fitted cuff matched to mid-arm circumference. Participants were instructed to avoid smoking, caffeine drinks and strenuous exercise for at least 30 min pre-measurement. Two readings were recorded at 1 min intervals; if the systolic difference was >10 mmHg, a third measurement was taken, with the average of the last two values adopted for analysis. Hypertension screening criteria (single-visit measurement, described as elevated blood pressure screening outcome, not definitive clinical diagnosis): Mean SBP ≥ 140 mmHg OR mean DBP ≥ 90 mmHg, OR self-reported regular antihypertensive medication use.

### 2.3. Principal Component Analysis (PCA)

Varimax orthogonal rotation PCA was conducted on standardized food consumption data of the 17 aggregated food groups. Key PCA diagnostic indicators: KMO statistic = 0.78, Bartlett’s sphericity test *p* < 0.001, confirming data suitability for factor extraction.

Retention criteria: Eigenvalue > 1.0 combined with scree plot elbow inflection point, retaining four independent dietary components. Of the 17 food groups entered into PCA, 15 had absolute factor loadings ≥ 0.45 on the four retained components and were considered salient contributors for pattern interpretation. Continuous pattern adherence scores were calculated for each participant by weighted factor regression; scores were split into four equal quartiles for stratified regression analysis. Total daily energy intake could not be quantified from the current FFQ design; therefore, energy adjustment was not implemented, acknowledged as a study limitation.

### 2.4. Statistical Analysis

Continuous variables were summarized as mean ± standard deviation, and categorical variables as frequencies and percentages. Group comparisons were performed using analysis of variance (ANOVA) for continuous variables and chi-square tests for categorical variables. To examine the associations between dietary patterns, physical activity, and hypertension, robust Poisson regression models were employed. All regression models adjusted for town-level sampling clusters to account for a multistage stratified survey design. Variance inflation factor (VIF) screening confirmed no severe multicollinearity (all VIF < 2.5). Three consistent adjustment models were applied uniformly for dietary patterns and physical activity outcomes. For the analysis of dietary pattern quartiles with hypertension screening prevalence, three adjustment models were applied: Model 1 was unadjusted; Model 2 was adjusted for age, sex, monthly household income, sleep duration, and family history of hypertension; Model 3 was Model 2 with additional adjustment for physical activity (mutual adjustment). For the analysis of physical activity tertiles with hypertension prevalence, Models 1 and 2 were identical, but Model 3 additionally adjusted for all four continuous dietary pattern scores (mutual adjustment). Mantel–Haenszel chi-square trend tests assessed linear dose–response trends across quartile/ordered activity tiers. Partial correlation analysis (adjusted for age, monthly income, number of children, sedentary time) evaluated linear associations between continuous dietary pattern scores and weekly MET-minutes. Two-sided *p* < 0.05 defined statistical significance; risk of false positive findings from multiple pattern/quartile testing is discussed in study limitations. All statistical analyses were conducted using SPSS version 28.0, and a two-sided *p*-value < 0.05 was considered statistically significant.

## 3. Results

### 3.1. Participant Demographics and Hypertension Prevalence

A total of 608 eligible adults were included in this study (356 males, 58.6%; 252 females, 41.4%). Participants were classified into three subgroups according to age strata: young adults (18–25 years) representing 16.1% of the sample, middle-aged adults (26–55 years) representing 58.2%, and older adults (55 years and above) representing 25.7%. The overall prevalence of hypertension was 24.34%. When disaggregated by sex, the prevalence was significantly higher among males at 29.8% compared with females at 16.7% (*p* < 0.001).

Table 1 displays all participant sociodemographic, lifestyle and hypertension status data. Adults with hypertension were markedly more prone to be male, older, married, self-employed, and to have attained only primary education (all *p* < 0.05). Religious affiliation, nightly sleep duration, and personal hypertension family history showed weaker or non-significant between-group differences. Physical activity tertile distribution did not differ significantly across hypertensive and normotensive groups in unadjusted chi-square testing (*p* = 0.109). These findings indicate that sociodemographic characteristics, particularly age, sex, and socioeconomic status, play a substantial role in the distribution of hypertension within this population.

### 3.2. Identification of Dietary Patterns

**Figure 1:**
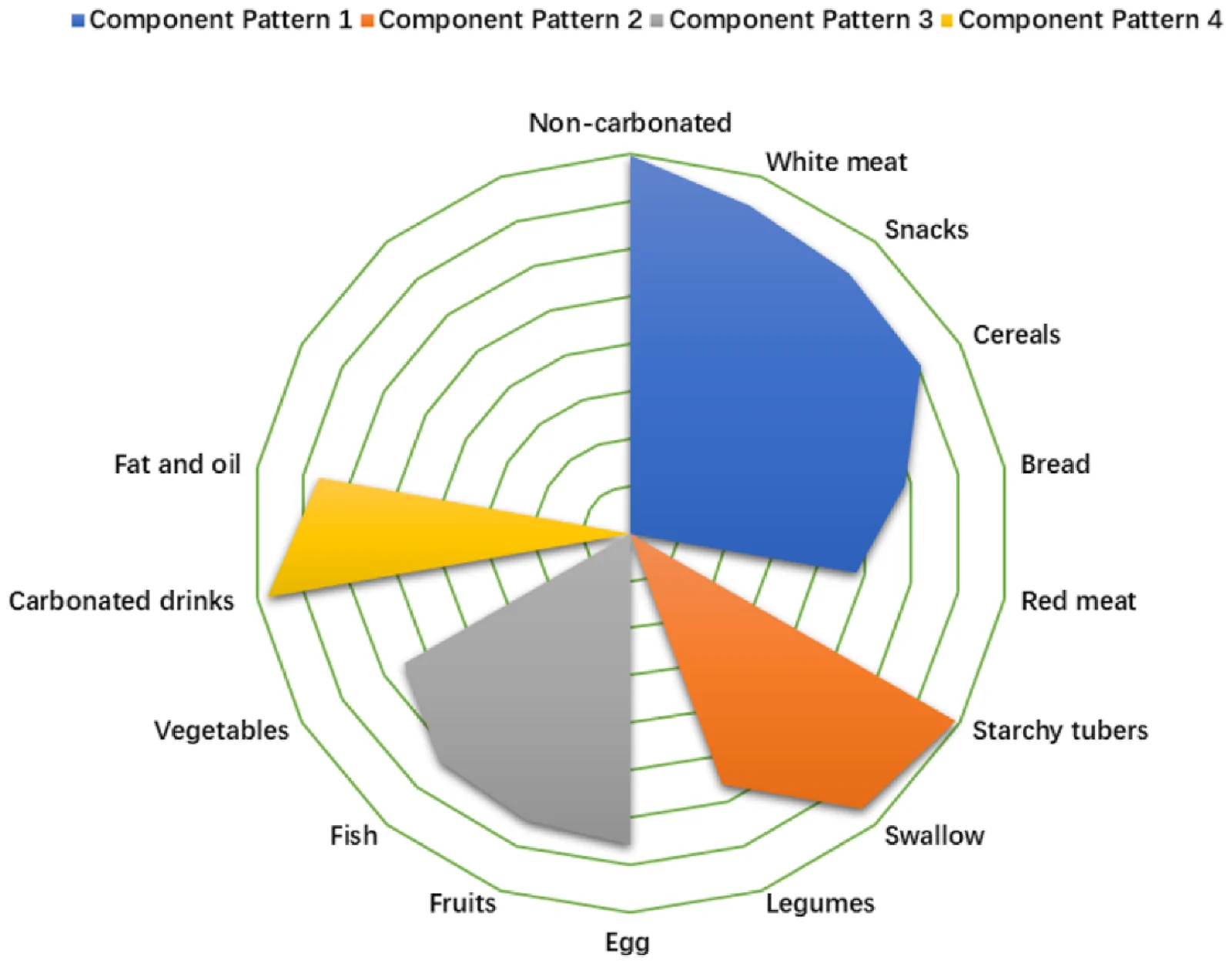
Radar chart visualizes factor loading distributions for four distinct dietary patterns derived from PCA. Note: Radar chart illustrating factor loadings for the four dietary patterns derived from Varimax-rotated PCA on 15 consolidated food groups. Each radial axis represents a food group, and the distance from the center reflects the loading magnitude.

**Figure 2:**
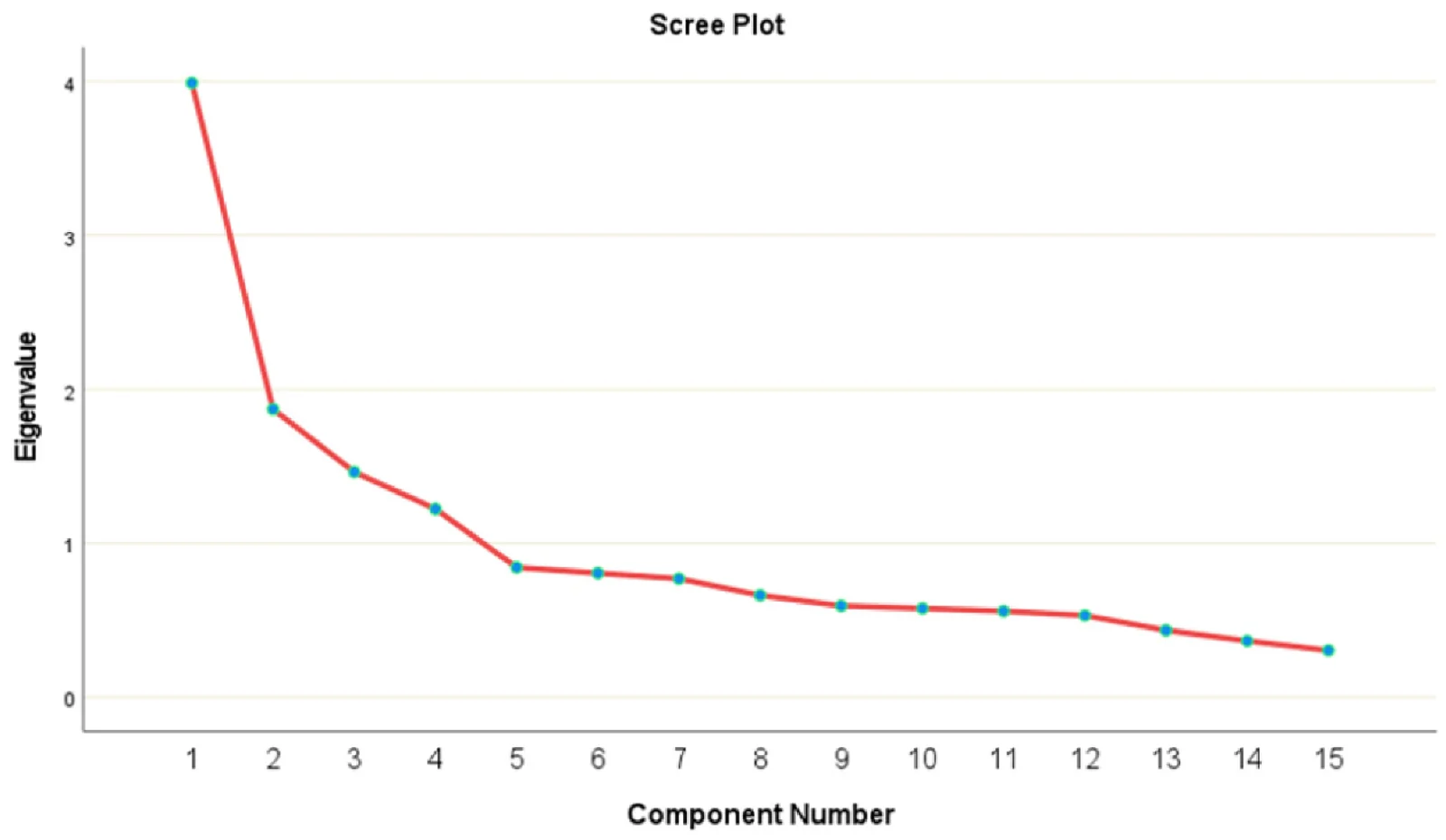
Scree plot of the PCA. Note: Eigenvalues > 1.0 combined with the elbow point on the curve to determine the number of components retained.

Dietary patterns among participants were derived using PCA, and the results are presented in Figure 1 and Table 2. Varimax-rotated PCA was applied to 100 FFQ food items. The analysis initially extracted 17 principal components, which were consolidated into 15 interpretable food groups after correlation and loading screening, from which four stable dietary patterns satisfying eigenvalue > 1.0 and scree plot criteria were retained (Figure 2). Collectively, the four patterns explained 57.1% of total variance in participant food consumption, representing a robust summary of habitual local dietary behavior. Individual pattern variance contributions were 26.6% (diversified traditional pattern), 12.5% (typical traditional pattern), 9.8% (fruits and vegetables pattern), and 8.2% (fats and soft drinks pattern). Table 2 reports full factor loadings for each food group against the four extracted patterns, with loadings below absolute 0.45 omitted for readability.

Pattern I (diversified traditional pattern): Defined by high consumption of non-carbonated beverages, white meat, savory snacks, cereals, bread, and moderate red meat intake.

Pattern II (typical traditional pattern): Dominated by starchy tubers, staple swallow meals, legumes, and moderate vegetable consumption prepared with local seasonings.

Pattern III (fruits and vegetables pattern): Characterized by regular intake of fresh vegetables, eggs, freshwater fish, and whole fruits.

Pattern IV (fats and soft drinks pattern): Centered on sugary carbonated sodas and added cooking oils/fats.

Collectively, PCA identified four distinct dietary patterns: diversified traditional, traditional, fruit and vegetable, and fat and soft drinks, explaining 57.1% of the total variance.

### 3.3. Association Between Dietary Patterns and Physical Activity

As shown in Table 3, after adjusting for age, monthly income, children counts and sedentary time, the partial correlation analysis indicated that Pattern I (diversified traditional pattern) and Pattern II (typical traditional) had positive correlations with physical activity (*r* = 0.114, *p* = 0.005; *r* = 0.106, *p* = 0.009), respectively. Pattern III (fruits and vegetables pattern) had a negative correlation with physical activity (*r* = −0.093, *p* = 0.020). All these correlation coefficients were small, suggesting weak correlations.

In brief, correlations were observed between dietary patterns and physical activity: Pattern I and Pattern II showed positive associations, while Pattern III showed a negative association; no significant correlation was found for Pattern IV (fats and soft drinks).

### 3.4. Association Between Dietary Patterns and Hypertension

The association between dietary patterns and hypertension was examined using robust Poisson regression analysis, with results presented in Table 4. The Mantel–Haenszel chi-square test revealed a significant linear trend between hypertension and Pattern II and Pattern III (*p* < 0.05). After full adjustment for age, sex, monthly household income, sleep duration, family history of hypertension, and physical activity (Model 3), participants in the highest adherence quartile (Q4) of Pattern II had a significantly higher prevalence of hypertension compared with those in the lowest quartile (Q1), with a prevalence ratio of 1.642 (95% CI: 1.069–2.252, *p* = 0.024). In contrast, higher adherence to Pattern III was associated with a significantly lower prevalence of hypertension (PR = 0.408, 95% CI: 0.258–0.646, *p* < 0.001). No significant associations with hypertension were observed for the Pattern I or Pattern IV.

Overall, the traditional pattern was positively associated with hypertension, while the fruit and vegetable pattern was inversely associated, with no significant associations for the diversified traditional pattern or the fat and soft drinks pattern.

### 3.5. Physical Activity and Hypertension Screening Prevalence

The relationship between physical activity level and hypertension was examined using robust Poisson regression analysis, and the findings are summarized in Table 5. The Mantel–Haenszel chi-square test indicated a significant linear trend between physical activity levels and hypertension status (*p* < 0.05), suggesting that changes in physical activity corresponded with differences in hypertension prevalence. In the unadjusted model (Model 1), higher levels of physical activity were associated with a higher prevalence of hypertension compared with the low-activity category (PR = 1.674, 95% CI: 0.993–2.822; *p* = 0.053), although this association did not reach statistical significance. After adjusting for potential confounders (Model 2), the association was slightly strengthened and became statistically significant (PR = 1.765, 95% CI: 1.056–2.950; *p* = 0.030). In the fully adjusted model (Model 3), which additionally controlled for all four dietary patterns, the association was attenuated and no longer statistically significant (PR = 1.590, 95% CI: 0.943–2.681; *p* = 0.082).

In summary, higher levels of physical activity were associated with an increased likelihood of hypertension in the unadjusted model; however, after mutual adjustment for all dietary patterns, this association was attenuated and no longer statistically significant.

## 4. Discussion

This cross-sectional study of 608 Nigerian adults from southwestern Nigeria identifies clear divergent cross-sectional associations between four locally derived PCA dietary patterns and hypertension screening prevalence, alongside an unexpected crude association between higher physical activity and elevated blood pressure. The overall hypertension prevalence of 24.3% aligns with regional subnational estimates for southwest Nigeria [3,21], with consistently higher detection rates among male participants. Four distinct PCA-derived dietary patterns capture the dominant habitual food consumption behaviors within the study sample, with independent, opposing links to hypertension prevalence observed for the starch-heavy traditional pattern and produce-rich fruits and vegetables pattern.

The elevated hypertension prevalence associated with the typical traditional dietary pattern (Pattern II) may be partly attributable to the broader dietary context in which these foods are typically consumed in southwestern Nigeria. While the factor loadings for this pattern reflect starchy tubers, swallow meals, legumes, and vegetables, these staples are frequently accompanied by soups and stews prepared with discretionary salt, seasoning cubes, and palm oil in local food practices, which may be linked to higher sodium intake. Although vegetables are generally protective [22], their preparation with excessive salt and fats may offset these benefits. The metabolic effects of refined carbohydrates, including rapid glycemic excursions, insulin resistance, and chronic inflammation, further contribute to elevated blood pressure.

Conversely, the inverse association of the fruit and vegetable pattern (Pattern III) is attributable to its richness in potassium, magnesium, dietary fiber, and bioactive compounds that promote vasodilation, sodium excretion, and reduced oxidative stress [23,24]. The neutral findings for Patterns I and IV likely reflect mixed nutritional compositions (Pattern I) or indirect pathways through weight gain rather than direct pressor effects (Pattern IV).

These findings are consistent with previous studies in African and non-African populations. The positive association between traditional, starch-based patterns and hypertension prevalence observed in our study has been reported in other West African settings [25,26], while the inverse relationship between fruit/vegetable intake and blood pressure aligns with meta-analyses demonstrating that high fruit and vegetable consumption lowers hypertension risk in prospective studies [22,25,27]. Our results also echo the DASH and Mediterranean diet evidence, confirming that protective dietary effects are observable even without fully adopting Western dietary models.

However, the unexpected positive association between physical activity and hypertension contrasts with the well-established protective role of exercise [28]^.^ Two population-specific explanations account for this discrepancy. First, reverse causation may operate: participants with early hypertension symptoms may voluntarily increase occupational labor or daily movement after unrecognized blood pressure elevation begins. Local working residents who experience persistent headaches, fatigue or mildly elevated blood pressure (unrecognized early hypertension) often increase daily physical labor or casual walking as self-perceived health improvement behavior without formal medical consultation. This self-initiated activity elevation could create the crude positive activity–hypertension correlation observed in cross-sectional data. We emphasize this remains an unvalidated conjecture requiring longitudinal verification.

Second, measured activity within this sample consists overwhelmingly of physically demanding occupational labor rather than structured recreational exercise. Extended work hours, chronic workplace psychosocial stress, and insufficient rest periods associated with manual labor offset the blood pressure-lowering benefits of movement seen in structured leisure activity interventions. After statistical adjustment for all dietary patterns, the activity–hypertension association weakens substantially and loses significance, suggesting that the observed crude association may be partly explained by differences in dietary habits rather than a direct or causal effect of physical activity.

These findings carry preliminary implications for public health. Specifically, this study focuses exclusively on community residents in Oyo and Kwara States, offering insights into region-specific dietary patterns and their relationship with hypertension. They highlight potential directions for culturally appropriate dietary interventions that may be associated with lower sodium intake, improved cooking practices, and higher fruit/vegetable consumption within the Nigerian context. The traditional dietary pattern, while culturally embedded, may be amenable to modification to preserve healthy components while reducing salt and unhealthy fats. This starch-heavy dietary pattern is a core component of the local diet. The fruit and vegetable pattern, which showed an inverse association with hypertension prevalence, may offer a feasible and acceptable pathway for hypertension prevention, as these foods are locally available and affordable. Additionally, we identified a nuanced interplay between physical activity and hypertension, with occupational physical activity potentially contributing to stress-related hypertension rather than providing cardiovascular benefits. Furthermore, the physical activity findings highlight the importance of promoting structured leisure-time exercise and addressing occupational stressors, rather than simply encouraging any form of activity. These findings underscore the need for public health interventions tailored to local contexts, such as promoting fruit and vegetable consumption, balancing traditional diets to reduce sodium and unhealthy fats, and encouraging stress-reducing leisure-time physical activities. These insights can inform national dietary guidelines and community-based program aimed at curbing the rising hypertension burden in Nigeria and similar low- and middle-income settings undergoing rapid nutritional and lifestyle transitions. By focusing on a non-urban Nigerian population, our study extends existing research and contributes localized evidence to guide effective hypertension prevention strategies.

Several limitations must be acknowledged. Firstly, the cross-sectional study design cannot establish temporality between dietary and activity exposures and elevated blood pressure, and no causal inference can be drawn from all observed associations. Secondly, single-visit blood pressure measurement cannot confirm a clinical hypertension diagnosis, only screening-level elevated blood pressure outcomes. Thirdly, key classic hypertension confounders (BMI, tobacco smoking, and alcohol consumption) were not collected during data collection, introducing residual confounding that may shift observed prevalence ratio magnitudes. Fourthly, all dietary and activity data rely on participant self-report, introducing inherent recall bias that may distort exposure measurement. In addition, recruitment was restricted to two southwestern Nigerian states, limiting generalizability to ethnic and geographic subgroups across northern, eastern, and southern coastal Nigeria. Exclusion criteria eliminated participants with acute non-hypertension chronic illness, creating mild selection bias in sample composition.

For future research, there are some recommendations. First of all, longitudinal prospective cohort studies are required to confirm directional relationships between dietary patterns and incident hypertension, eliminating cross-sectional temporal ambiguity. In addition, subsequent data collection should integrate objective wearable physical activity monitoring to replace self-reported GPAQ data, alongside standardized anthropometric BMI measurement and validated alcohol and tobacco use surveys to capture all major confounding risk factors. Multi-state sampling covering all six Nigerian geopolitical zones would enable national-level dietary pattern comparison, generating nationally representative evidence for federal public health policy design. Controlled dietary intervention trials testing modified traditional low-sodium, high-produce meals would further validate the causal protective effects of fruit and vegetable consumption within local cultural eating contexts.

## 5. Conclusions

In conclusion, this PCA-based cross-sectional analysis of southwestern Nigerian adults identifies two dietary patterns with consistent independent associations with hypertension prevalence. A starch-heavy typical traditional dietary pattern is associated with higher hypertension prevalence after full covariate adjustment, while a diet abundant in fresh fruits and vegetables is associated with lower hypertension prevalence. Crude associations between elevated physical activity and greater hypertension prevalence disappear following mutual adjustment for all dietary patterns, although alternative explanations such as occupational labor stress or reverse causation cannot be ruled out and should be explored in future research.

These region-specific nutritional findings fill a critical local research gap and provide evidence to design culturally tailored hypertension prevention programs for Nigerian adults. Future prospective research incorporating complete confounding risk factor measurement and objective activity tracking is needed to confirm causal dietary pathways and refine national public health intervention strategies targeting diet-related cardiovascular disease burden across West Africa.

## Figures and Tables

**Table 1:** Sociodemographic and lifestyle characteristics stratified by hypertension status.

Variables	Total	Blood Pressure *n* (%)		*p*
		Normotension *n* (%)	Hypertension *n* (%)	
Gender				**<0.001**
Male	356	250 (70.2)	106 (29.8)	
Female	252	210 (83.3)	42 (16.7)	
Age				**<0.001**
Young adults	98	94 (95.9)	4 (4.1)	
Middle-aged adults	354	271 (76.6)	83 (23.4)	
Older adults	156	95 (60.9)	61 (39.1)	
Religion				**0.014**
Christian	260	193 (74.2)	67 (25.8)	
Islam	341	260 (76.2)	81 (23.8)	
Tradition	7	7 (100.0)	0 (0.0)	
Marital Status				**<0.001**
Single	208	175 (84.1)	33 (15.9)	
Married	367	259 (70.6)	108 (29.4)	
Divorced	14	12 (85.7)	2 (14.3)	
Widowed	19	14 (73.7)	5 (26.3)	
No. of Children				**<0.001**
None	205	169 (82.4)	36 (17.6)	
1–3	243	194 (79.8)	49 (20.2)	
4–6	132	82 (62.1)	50 (37.9)	
More than 6	28	15 (53.6)	13 (46.4)	
Occupation				**<0.001**
Self-employed	388	275 (70.9)	113 (29.1)	
Employed	150	119 (79.3)	31 (20.7)	
Unemployed	15	15 (100.0)	0 (0.0)	
Student	40	39 (97.5)	1 (2.5)	
Retired	15	12 (80.0)	3 (20.0)	
Education				**<0.001**
Primary	53	27 (50.9)	26 (49.1)	
Secondary	197	157 (79.7)	40 (20.3)	
Tertiary	358	276 (77.1)	82 (22.9)	
Family Type				0.217
Monogamous	501	384 (76.6)	117 (23.4)	
Polygamous	107	76 (71.0)	31 (29.0)	
Monthly Income				0.076
Less than ₦20,000	61	51 (83.6)	10 (16.4)	
₦20,000–50,000	215	169 (78.6)	46 (21.4)	
More than ₦50,000	332	240 (72.3)	92 (27.7)	
Sleep Duration				0.740
Less than 7 h	158	118 (74.7)	40 (25.3)	
More than 7 h	450	342 (76.0)	108 (24.0)	
Family History of Hypertension				0.077
Yes	119	87 (73.1)	32 (26.9)	
No	262	210 (80.2)	52 (19.8)	
Do not know	227	163 (71.8)	64 (28.2)	
Physical Activity				0.109
Low	302	238 (78.8)	64 (21.2)	
Moderate	275	202 (73.5)	73 (26.5)	
High	31	20 (64.5)	11 (35.5)	

Note: Categorical variables are presented as *n* (%). Between-group comparisons were conducted using the chi-square test; sparse zero cells adopted Fisher’s exact test. The bold *p* < 0.05 indicates statistical significance.

**Table 2:** Factor loadings and dietary patterns for 15 food groups obtained by factor analysis.

Food Groups	Pattern I	Pattern II	Pattern III	Pattern IV
Non-carbonated drinks	0.795			
White meat	0.735			
Snacks	0.713			
Cereals	0.704			
Bread	0.587			
Red meat	0.483			
Starchy tubers		0.791		
Swallow		0.757		
Legumes		0.562		
Eggs			0.656	
Fruits			0.643	
Fish			0.627	
Vegetables		0.458	0.551	
Soda/soft drinks				0.776
Fat oil				0.666

Note: PCA (principal component analysis) was used for this analysis. Factor analysis loading of <0.45 in absolute terms was excluded for simplicity; Pattern I: diversified traditional pattern; Pattern II: traditional pattern; Pattern III: fruit and vegetable pattern; Pattern IV: fat and soft drinks pattern.

**Table 3:** Partial correlation between dietary pattern scores and physical activity.

Dietary Pattern	Physical Activity	
	r	*p*
Pattern I	0.114	**0.005**
Pattern II	0.106	**0.009**
Pattern III	−0.093	**0.020**
Pattern IV	0.030	0.455

Note: This analysis adjusted for age, monthly income, number of kids and sedentary time. Pattern I: diversified traditional pattern; Pattern II: traditional pattern; Pattern III: fruit and vegetable pattern; Pattern IV: fat and soft drinks pattern. The bold *p*-value means *p* < 0.05.

**Table 4:** Robust cluster-adjusted Poisson regression linking dietary pattern quartiles with hypertension screening prevalence.

Dietary Pattern	Hypertension *n* (%)		*p*	Model 1 PR (95%CI)	*p*	Model 2 PR (95%CI)	*p*	Model 3 PR (95%CI)	*p*
	No	Yes							
Pattern 1									
Q1	110 (72.4)	42 (27.6)	0.167	1		1		1	
Q2	111 (73.0)	41 (27.0)		1.112 (0.768–1.610)	0.574	1.269 (0.880–1.830)	0.202	1.231 (0.854–1.774)	0.266
Q3	125 (82.2)	27 (17.8)		0.831 (0.534–1.293)	0.413	1.010 (0.650–1.568)	0.965	0.976 (0.631–1.511)	0.913
Q4	114 (75.0)	38 (25.0)		0.789 (0.543–1.149)	0.216	0.946 (0.624–1.433)	0.793	0.892 (0.589–1.351)	0.590
Pattern 2									
Q1	128 (84.2)	24 (15.8)	**<0.001**	1		1		1	
Q2	124 (81.6)	28 (18.4)		1.179 (0.717–1.938)	0.516	1.032 (0.633–1.681)	0.900	1.014 (0.624–1.646)	0.955
Q3	107 (70.4)	45 (29.6)		2.035 (1.318–3.141)	**0.001**	1.577 (1.027–2.423)	**0.038**	1.522 (0.989–2.344)	**0.056**
Q4	101 (66.4)	51 (33.6)		2.274 (1.479–3.496)	**<** **0.001**	1.754 (1.148–2.680)	**0.009**	1.642 (1.069–2.252)	**0.024**
Pattern 3									
Q1	109 (71.7)	43 (28.3)	**0.012**	1		1		1	
Q2	112 (73.7)	40 (26.3)		0.884 (0.619–1.264)	**0.500**	0.399 (0.252–0.630)	**<0.001**	0.805 (0.569–1.138)	0.220
Q3	109 (71.7)	43 (28.3)		0.953 (0.665–1.366)	0.793	0.876 (0.624–1.229)	0.443	0.886 (0.631–1.242)	0.482
Q4	130 (85.5)	22 (14.5)		0.476 (0.300–0.754)	**0.002**	0.804 (0.570–1.135)	0.215	0.408 (0.258–0.646)	**<0.001**
Pattern 4									
Q1	114 (75.0)	38 (25.0)	0.975	1		1		1	
Q2	114 (75.0)	38 (25.0)		0.992 (0.676–1.455)	0.965	0.991 (0.679–1.444)	0.953	0.968 (0.663–1.414)	0.962
Q3	117 (77.0)	35 (23.0)		0.902 (0.656–1.393)	0.606	0.986 (0.677–1.437)	0.941	0.991 (0.690–1.424)	0.889
Q4	115 (75.7)	37 (24.3)		0.956 (0.656–1.393)	0.814	1.011 (0.705–1.405)	0.961	0.991 (0.690–1.424)	0.868

Note: Q1 = lowest adherence quartile (reference); Model 1: Unadjusted; Model 2: Adjusted for age, sex, monthly household income, sleep duration, and family history of hypertension; Model 3: Model 2 with additional adjustment for physical activity (mutual adjustment). Mantel–Haenszel trend *p*-values listed at table footnotes. Bold *p* < 0.05.

**Table 5:** Analysis of the association between physical activity tertiles and hypertension prevalence.

Variables	Hypertension *n* (%)		*p*	Model 1 PR (95%CI)	*p*	Model 2 PR (95%CI)	*p*	Model 3 PR (95%CI)	*p*
	No	Yes							
Physical Activity									
Low	238(78.8)	64(21.2)	**0.004**	1		1		1	
Moderate	202(73.5)	73(26.5)		1.253 (0.934–1.679)	0.132	1.350 (1.011–1.802)	**0.042**	1.220 (0.905–1.644)	**0.192**
High	20(64.5)	11(35.5)		1.674 (0.993–2.822)	**0.053**	1.765 (1.056–2.950)	**0.030**	1.590 (0.943–2.681)	**0.082**

Note: The chi-square trend test and robust Poisson regression analysis were used for analysis. Q1 was the reference group. Model 1: Unadjusted; Model 2: Adjusted for age, sex, monthly household income, sleep duration, and family history of hypertension; Model 3: Model 2 with additional adjustment for all four continuous dietary pattern scores (mutual adjustment). The bold *p*-value means < 0.05.

## Data Availability

The datasets generated and analyzed during the current study are not publicly available due to ethical restrictions but are available from the corresponding author on reasonable request.

## Supplementary Materials

The following supporting information can be downloaded at: https://www.mdpi.com/article/10.3390/nu18172825/s1, Table S1: Full grouping scheme of the 100-item culturally adapted FFQ.
