# Supplementary material for: Cross-Sectional Associations of Dietary Pattern, Physical Activity with Hypertension Among Adults in Southwestern Nigeria
Source: Nutrients. 2026 Aug 28;18(17):2825. doi: 10.3390/nu18172825 (PMC13566991; doi:10.3390/nu18172825)
Supplement: Supplementary file 1 [file nutrients-18-02825-s001.zip › nutrients-4479802-supplementary.pdf]

Supplemental materials

Supplementary Table S1: Full grouping scheme of the 100-item culturally adapted FFQ

Serial number of single food item	Name of each individual food	Assigned preliminary broad food category	Brief classification basis
1	Semo	Swallow-based foods	Starchy staple, eaten with soups
2	Eba		
3	Amala		
4	Wheat		
5	Fufu		
6	Poundo yam		
7	Semovita		
8	Beans	Legumes and legume-based dishes	Plant protein, dietary fiber
9	Gbegiri		
10	Akara		
11	Ekuru		
12	Moimoi		
13	Beans Porridge		
14	Yam	Starchy roots and tubers	Carbohydrate-rich, energy source
15	Yam porridge		
16	Cocoyam		
17	Sweet potatoes		
18	Irish Potatoes		
19	Cassava		
20	Cassava flakes (Garri)		
21	Spaghetti / Pasta	Cereals and cereal products	Refined carbohydrate, low fiber
22	Corn pap		
23	Eko		
24	Oats		
25	Golden Morn		
26	Corn Flakes		
27	Porridge		
28	White bread	Breads	Cereal product, energy source
29	Coconut bread		
30	Banana bread		
31	Wheat bread		
32	Sardine bread		

Serial number of single food item	Name of each individual food	Assigned preliminary broad food category	Brief classification basis
33	White rice	Rice and rice-based dishes	Staple grain, energy source
34	Jollof rice		
35	Fried rice		
36	Ofada rice		
37	Jambalaya rice		
38	Coconut rice		
39	Dirty rice		
40	Native rice		
41	Beef	Red meat	Animal protein, saturated fat, iron
42	Pork		
43	Chevon (Goat meat)		
44	Suya		
45	Chicken	White meat	Low-fat animal protein
46	Turkey		
47	Pawpaw	Fruits	Vitamins, minerals, dietary fiber
48	Watermelon		
49	Pineapple		
50	Guava		
51	Cucumber		
52	Avocado Pear		
53	Mangoes		
54	Banana		
55	Coconut		
56	Agbalumo (African cherry)		
57	Apples		
58	Orange		
59	Tangerine		
60	Grapefruit		
61	Carrot	Vegetables	Vitamins, fiber, minerals
62	Garden Egg		
63	Cabbage		
64	Pepper and tomato Stew		
65	Okro		

Serial number of single food item	Name of each individual food	Assigned preliminary broad food category	Brief classification basis
66	Corn		
67	Pumpkin		
68	Mushroom		
69	Vegetable Soup (Efo Riro)		
70	Ewedu		
71	Egusi		
72	Butter	Oil and fats	Essential fatty acids, energy
73	Mayonnaise		
74	Vegetable oil		
75	Palm oil		
76	Fish	Fish	Omega-3 fatty acids, high-quality protein
77	Chicken eggs	Eggs	High-quality protein, choline
78	Cream Milk	Milk and dairy products	Calcium, animal protein
79	Soya Milk		
80	Yoghurt		
81	Ice Cream		
82	Powdered Milk		
83	Tea	Beverages	Beverages, phytochemicals
84	Coffee		
85	Bournvita / Milo		
86	Fruit juice		
87	Coca-Cola	Carbonated soft drinks	Added sugar, empty calories
88	Fanta		
89	Pepsi		
90	Malt drinks		
91	Cake	Confectionaries and snacks	High sugar/fat, energy-dense
92	Puff Puff / Buns / Donuts		
93	Chin-chin		
94	Shawarma		
95	Pizza		
96	Burger		

Serial number of single food item	Name of each individual food	Assigned preliminary broad food category	Brief classification basis
97	Chocolate Bar		
98	Meat Pie		
99	Fish Pie		
100	Pop Corn		

Note: All 100 food entries were manually classified into 17 broad food
